# Cost-Utility Analysis of Thumb Replantation in Working Patients Aged Between 65-70 in China

**DOI:** 10.1093/geroni/igaf122.2164

**Published:** 2025-12-31

**Authors:** Jing Bai

**Affiliations:** Yale University, New Haven, Connecticut, United States

## Abstract

Thumb replantation plays a critical role in restoring hand function after traumatic amputation, significantly impacting grip strength, dexterity, and quality of life. While typically recommended for younger patients, its utility in actively working individuals aged 65–70 remains underexplored. This study evaluates the cost-utility of thumb replantation compared to revision amputation in this demographic in China, focusing on economic and functional outcomes. A decision-tree-based cost-effectiveness analysis was conducted from a societal perspective using data from Shanghai Jiao Tong University Affiliated Sixth People’s Hospital. Patients aged 65–70 who underwent thumb replantation or revision amputation were included. The primary outcome was the incremental cost-effectiveness ratio (ICER), with functional recovery assessed via Disabilities of the Arm, Shoulder, and Hand (DASH) score and total active motion (TAM) degree. Cost data included surgical, rehabilitation, and indirect costs (e.g., lost wages). Sensitivity analyses (one-way and probabilistic) were performed to assess the robustness of the results. Replantation demonstrated superior functional outcomes with lower DASH scores (mean 25.31 vs. 29.75) and higher TAM degrees (mean 106.78 vs. 44.62) compared to revision amputation (both p < 0.001). Replantation also incurred lower total costs (183,195.36 CNY vs. 198,055.67 CNY). The ICER for replantation was -3346.28 CNY per unit improvement in DASH score and -239.08 CNY per unit improvement in TAM degree, indicating cost savings alongside better outcomes. Thumb replantation is a cost-effective intervention for actively working elderly patients in China, offering superior functional outcomes and lower overall costs compared to revision amputation.

